# Lipoprotein(a) and Aortic Valve Stenosis: From Pathophysiology to Emerging Pharmacological Agents

**DOI:** 10.3390/jcm15010274

**Published:** 2025-12-30

**Authors:** Federica Agnello, Giulia Laterra, Lorenzo Scalia, Maria Sara Mauro, Orazio Strazzieri, Claudia Reddavid, Salvatore Ingala, Simona Guarino, Chiara Barbera, Maria Daniela Russo, Marco Barbanti

**Affiliations:** 1 Faculty of Medicine and Surgery, Università degli Studi di Enna “Kore”, Piazza dell’Università, 94100 Enna, Italy; 2 Division of Cardiology, Ospedale Umberto I, ASP 4 di Enna, 94100 Enna, Italy; 3 Division of Cardiology, A.O.U. Policlinico “G. Rodolico San Marco”, 95123 Catania, Italy

**Keywords:** Lp(a), aortic valve stenosis, atherosclerosis, PCSK9i, statin

## Abstract

Aortic valve stenosis (AVS) is the most common valvular disease in developed countries, and no pharmacological therapy is currently available. Increasing evidence identifies lipoprotein(a) [Lp(a)] as a causal factor linking lipid metabolism, inflammation, and valve calcification. Lp(a) levels are largely genetically determined and remain stable throughout life, making them a potential therapeutic target. This review summarizes the current evidence on Lp(a) and AVS pathophysiology, the diagnostic and prognostic role of Lp(a), and the therapeutic potential of Lp(a)-lowering agents. Emerging Lp(a)-targeted therapies, including antisense oligonucleotides and siRNA-based agents, could reshape AVS management by providing the first pharmacological option to slow disease progression in selected high-risk patients.

## 1. Introduction

Aortic valve stenosis (AVS) is the most common valvular heart disease in developed countries (with a prevalence of approximately 40% in those over 75 years) and its prevalence is expected to increase, due to longer life expectancy [1]. Symptomatic AVS has poor prognosis, and the only available treatment options are surgical aortic valve replacement (SAVR) and transcatheter aortic valve implantation (TAVI), both recommended as class I, level A indications in latest guidelines for the management of valvular heart disease of the European Society of Cardiology and the European Association for Cardio-Thoracic Surgery (ESC/EACTS) [2]. Degenerative calcific AVS is the most common cause of AVS, sharing common pathophysiology with atherosclerosis [3,4,5].

Conversely, no modifiable risk factor has been identified as a potentially therapeutic target to slow AVS progression. Therefore, patients with mild or moderate aortic stenosis undergo periodic follow-up visits, since effective pharmacological options are currently lacking [6,7].

In this context, lipoprotein(a) [Lp(a)] has emerged as a novel risk factor, playing a determinant role in the development of cardiovascular disease, including coronary artery disease (CAD), aortic valve calcification, and stenosis [8,9]. In fact, recent research has shown that Lp(a) promotes atherosclerosis, inflammation, and thrombosis [10]. Regarding the link between Lp(a) and AVS, many observational studies supported its association with aortic valve calcification and AVS progression; next, large Mendelian randomization studies revealed the causal role of elevated Lp(a) levels in higher incidence of aortic valve calcifications and AVS [11,12,13,14,15,16,17].

This evidence has paved the way to a new field of research, investigating pharmacological strategies to reduce Lp(a) concentration and address different cardiovascular unmet clinical needs, such as the management of residual cardiovascular risk and the progression of AVS [18].

**Figure 1:**
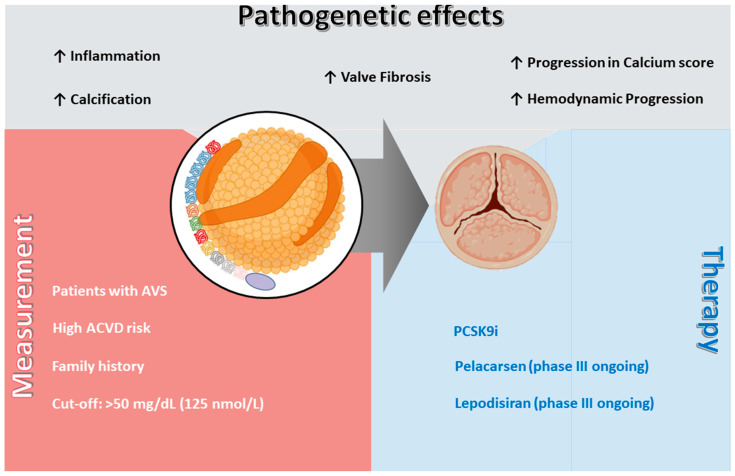
**[Lp(a)] and aortic valve stenosis (AVS); pathogenetic effects, measurement indications, and therapeutic perspectives.** Lp(a) contributes to AVS development and progression through multiple mechanisms, including inflammation, valve fibrosis, calcification, and hemodynamic progression, as well as acceleration of the aortic valve calcium score. Measurement of Lp(a) is recommended in patients with AVS, in those with high risk of ASCVD, and in individuals with a family history of premature ASCVD, with a suggested cut-off value of >50 mg/dL (125 nmol/L). Currently available therapies include PCSK9i, while novel specific agents such as pelacarsen and lepodisiran are under evaluation in ongoing phase III trials. **Abbreviations:** AVS = aortic valve stenosis; ASCVD = atherosclerotic cardiovascular disease; Lp(a) = lipoprotein(a); PCSK9i = proprotein convertase subtilisin/kexin type 9 inhibitors.

This review aims to explore the pathophysiological link between AVS and Lp(a), summarize the evidence connecting Lp(a) with AVS, examine both available and emerging therapeutic approaches (Figure 1).

This is a narrative review based on a comprehensive, though non-systematic, search of the literature. Relevant publications were identified through searches in PubMed, Scopus, and Web of Science databases using combinations of the keywords “lipoprotein(a)”, “aortic valve stenosis”, “calcific aortic valve disease”, and “atherosclerosis”. The search covered studies published between 2000 and 2025, with no language restrictions applied, although most sources were in English. Additional relevant papers were retrieved from the reference lists of key articles and from the authors’ own expertise in the field. Studies were selected for inclusion based on their relevance to the pathophysiology, clinical associations, and therapeutic implications of Lp(a) in AVS

## 2. Biochemical Structure and Metabolism of Lp(a)

Lp(a) is a lipid particle with a biochemical structure very similar to low-density lipoprotein cholesterol (LDL-C). The particle is primarily synthesized in the liver, without significant influence by environmental or dietary factors. In fact, its plasma concentration is mostly genetically determined by the LPA gene, located on chromosome 6q26–27.

Lp(a) consists of a cholesterol-rich lipid core surrounded by a molecule of apolipoprotein B100 (apo B100), as well as LDL, with the addition of apolipoprotein A (Apo A) covalently linked by a disulfide bridge [19].

Apo(a) shares significant relevant homology with plasminogen, reflecting a likely evolutionary relationship between their respective coding genes. Nevertheless, despite the structural similarities with plasminogen, Lp(a) plays a prothrombotic effect, preventing plasminogen conversion into plasmin and favoring plasmin production. Lp(a) is composed of multiple highly glycosylated looped domains, known as kringles (because of their repetitive structure) and an inactive protease-like domain. Specifically, apo(a) contains one kringle type V (KV) domain, and 10 types of kringle IV (KIV) domains, each present as a single copy—except for KIV type 2, which is present in a variable number of tandem repeats (ranging from fewer than 10 to over 40 copies) [19]. This variability in KIV-2 repeats number determines the size heterogeneity of apo(a) and it is inversely associated with plasma Lp(a) concentrations. In fact, subjects with smaller Lp(a) isoforms, characterized by fewer KIV2 repeats, typically show higher plasma concentrations of the particle and a greater cardiovascular risk, likely due to increased hepatic production rates [20].

The number of KIV2 repeats is only one of the genetic determinants of Lp(a) levels; in fact, the heritability accounts for 70% to over 90% [19]. Several single-nucleotide polymorphisms (SNPs) independently modulate Lp(a) levels, identified both at the LPA locus and in the APOE and APOH genes [21].

Indeed, Lp(a) plasma levels are determined by many mechanisms, including both isoform-dependent and independent ones. Smaller apo(a) isoforms are more efficiently secreted by hepatocytes, while larger isoforms are more prone to intracellular retention and proteasomal degradation, which further explains the inverse relationship between isoform size and Lp(a) concentration [22]. Additionally, variation in LPA gene expression, differences in mRNA transcript stability, and translation efficiency may also contribute to Lp(a) plasma level [22].

The catabolism of the molecule takes place mainly in the liver, although the receptors involved have not been completely identified [23,24]. While the LDL receptor (LDLR) may contribute under specific conditions—such as during combined treatment with statins and proprotein convertase subtilisin/kexin type 9 inhibitors (PCSK9i)—its physiological role in Lp(a) metabolism appears limited. In fact, kinetic studies have shown comparable clearance rates of radiolabeled Lp(a) in individuals with and without functional LDLRs, including those with familial homozygous LDLR deficiency [25]. Alternative receptors have been hypothesized to participate in Lp(a) uptake. Among these, plasminogen receptors, such as Plg-RKT, have attracted interest due to structural similarities between apo(a) and plasminogen, though direct human evidence remains lacking [26]. Other hepatic scavenger receptors, including SR-B1 and members of the LDLR–related protein family (LRP1 and LRP8), may also be involved, but their precise contribution to Lp(a) turnover in humans has yet to be clearly established [24].

Emerging data suggest that the biochemical composition of Lp(a)—including apo(a) isoform size, lipid cargo, and associated apolipoproteins—could influence receptor selectivity and catabolic efficiency. For instance, the presence of accessory proteins such as ApoH, ApoC-III, and ApoE on the Lp(a) surface may modulate its interaction with clearance pathways, although these findings are still under active investigation [27].

Importantly, Lp(a) levels are largely stable throughout life, generally established by the age of five, and are only minimally influenced by environmental or lifestyle factors [28]. Nonetheless, biological variability of up to 20% has been reported in serial measurements, suggesting that repeated testing may be warranted in certain clinical contexts to improve cardiovascular risk stratification and explains a significant portion of the interindividual variability [29].

Finally, ancestry significantly influences Lp(a) plasma concentrations and isoform distribution. Individuals of African and South Asian descent generally exhibit higher levels compared to those of European or East Asian ancestry [28]. Despite these differences, the relationship between elevated Lp(a) and cardiovascular risk appears consistent across populations, supporting the use of unified diagnostic thresholds.

## 3. The Aortic Valve: From Endothelial Dysfunction to Calcific Stenosis

The preclinical manifestation of aortic valve degeneration, is represented by aortic sclerosis, affecting a quarter of individuals over 65 years and half of those over 85 years of age [30]. The initial lesion is the focal calcification without alterations in gradients and velocity.

**Figure 2:**
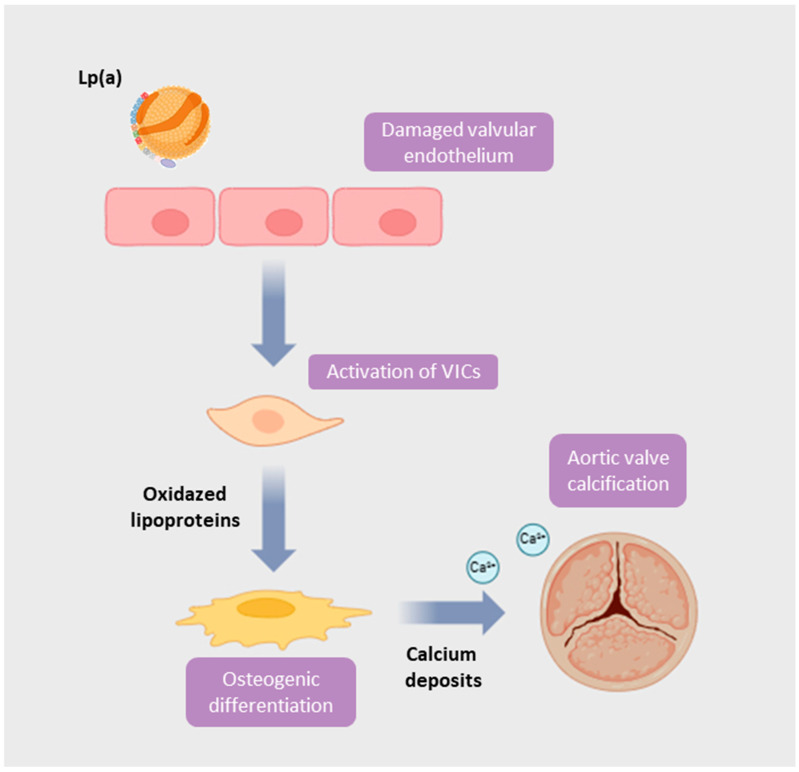
**Mechanism linking lipoprotein(a) [Lp(a)] to aortic valve calcification.** Elevated Lp(a) contributes to endothelial dysfunction and lipid infiltration in the aortic valve, leading to activation of valve interstitial cells (VICs). Oxidized phospholipids derived from Lp(a) promote VIC osteogenic differentiation and calcium deposition, ultimately driving progressive valvular calcification and stenosis.

Calcific AVS is the result of a multifactorial process involving lipid accumulation, cell apoptosis, and activation of osteogenic pathways, upon an inflammatory background. Within this intricate network of cellular and molecular interactions, Lp(a) arises as a pro-inflammatory and pro-atherogenic determinant, which contributes to the development of this pathological condition (Figure 2).

As in atherosclerosis, endothelial dysfunction caused by shear stress represents a key moment in the pathogenesis of calcific AVS, since the endothelium of the aortic valve plays a protective role against valve degeneration, in a multi-modal way. The endothelial injury triggers inflammation and lipid infiltration in the aortic valve cusps’ inner layers. Moreover, the loss of the endothelium integrity alters the normal expression of anti-osteogenic genes and inhibits endothelial nitric oxide synthase (eNOS) pathway that normally prevents the differentiation of vascular smooth muscle cells into osteoblastic ones.

Lp(a) begins to exert its pathogenetic contribution already during the initial phase with lipid infiltration. In the inner layers of valve cusps, reactive oxygen species initiate a cascade of lipid modification, starting with the formation of oxidized Lp(a) and oxidized LDL, and finally culminating with their lysophospholipid derivatives. In particular, lysophosphatidylcholine, resulting from oxidized Lp(a), induces a crucial phase of the AVS pathogenesis, characterized by the apoptosis of valve interstitial cells and the upregulation of adhesion molecules and of bone morphogenetic protein (BPM2, BPM6) involved in ectopic osteogenic signaling, which lead to valve mineralization. Indeed, the process of mineralization and osteogenesis is amplified by the inflammatory infiltrate, rich in macrophages, monocytes, mast cells, and T cells. In fact, the endothelial expression of adhesion molecules, as well as vascular cell adhesion molecule-1, allows these cells to adhere and infiltrate the valvular sub-endothelium, where they differentiate in macrophages and activated T cells and release growth factors and pro-inflammatory cytokines such as interleukin-1 (IL-1) and tumor necrosis factor alpha (TNF-α), thus promoting fibrosis and calcification. In this context, macrophages and valvular interstitial cells (VICs) play a bidirectional link, creating a pro-inflammatory and pro-calcific microenvironment. On the one hand, macrophages produce inflammatory cytokines which stimulate VIC to differentiate into osteoblastic-like cells; on the other hand, activated VICs promote fibrosis by releasing Transforming growth factor beta (TGF-β) and reducing MMP-9 activity in macrophages, leading to increased collagen deposition. Moreover, inflammation also stimulates pathological angiogenesis processes contributing to the progression of calcific valve degeneration Another potent activator of this pathologic remodeling is angiotensin-converting enzyme, which stimulates collagen production through angiotensin II, which is the rationale of Renin-angiotensin system blockade therapy [31].

Recent advances in molecular profiling have further expanded our understanding of the mechanisms linking Lp(a) to aortic valve calcification. Single-cell transcriptomics has enabled the identification of novel potential therapeutic targets in aortic valve disease, particularly those modulated by lipoprotein(a) and its oxidized phospholipids. Among these, autotaxin (ATX) has emerged as a key enzyme that metabolizes lysophospholipids associated with Lp(a) and is found to be overexpressed in calcified valvular interstitial cells; notably, ATX inhibition has been associated with reduced valvular mineralization [32]. Another promising target is represented by oxidized phospholipids (OxPL) themselves, which promote osteogenic differentiation of VICs and the progression of calcification. Strategies aimed at neutralizing OxPL, such as the use of monoclonal antibodies, are currently under investigation [13,17].

In addition, transcriptomic analyses have highlighted the activation of inflammatory pathways, including those mediated by interleukin-6 (IL-6) and the NLRP3 inflammasome, which are triggered by lipid deposition and contribute to the osteoblastic transformation of valvular cells [32,33]. Finally, modulation of NOTCH1 signaling and nitric oxide pathways, both involved in regulating valvular calcification and fibrosis, represents another promising therapeutic avenue [33].

The first observational studies suggesting a potential link between elevated serum levels of Lp(a) and the risk of AVS date back to the late 1990s [32]. Nevertheless, it was only in the following decades that large-scale prospective studies and genetic analyses provided stronger evidence. Early insights were provided by Arsenault et al., who analyzed over 17,000 participants from the EPIC- (European Prospective Investigation into Cancer) Norfolk cohort, revealing a significantly increased risk of calcific AVS among individuals in the highest tertile of Lp(a), even after adjusting for conventional cardiovascular risk factors [17]. These findings were corroborated by a larger study across two large population-based cohorts that showed Lp(a) concentration above the 95th percentile was associated with a nearly threefold increased risk of calcific AVS (95% CI: 1.8 to 4.9) [13].

On a genetic basis, specific SNP within the locus of LPA gene have been linked to AVS. The most consistently implicated variant is rs10455872, which reached genomewide significance for association with aortic valve calcification and incidence of AVS, in a study involving 6942 individuals [33]. Further investigations found out a greater genetic risk of AVS among individuals carrying the rs3798220 variant as well [34].

While the relation of Lp(a) and the incidence of AVS has been largely established, evidence regarding the role of Lp(a) in the progression of AVS is weaker and debatable. A secondary analysis of the ASTRONOMER (Aortic Stenosis Progression Observation: Measuring the Effects of Rosuvastatin) trial, including 220 patients with mild-to-moderate AVS echocardiographically followed up for 3–5 years, showed a linear association between Lp(a) serum concentration and faster progression of calcific AVS (odds ratio [OR] per 10 mg/dL increase, 1.10; 95% CI, 1.03–1.19; *p* = 0.006), especially in younger patients (OR for Lp[a] level per 10 mg/dL increase, 1.19 [95% CI, 1.07–1.33; *p* = 0.002] [17]. A recent meta-analysis including data from 757 individuals, showed that patients with the highest Lp(a) level present a faster progression of peak aortic jet velocity and mean transvalvular gradient [15]. Similar results emerged in another study including 145 patients, showing that Lp(a) significantly drives AVS progression, using both computed tomography and echocardiography [14]. On the contrary, a study of 922 patients showed Lp(a) was associated with new onset of aortic valve calcium but not with aortic valve calcium progression (β: −71 AU for each 50 mg/dL higher Lp(a); 95% CI −117; 35) [16]. These results are aligned with the analysis of the MESA study (Multi-Ethnic Study of Atherosclerosis), which found that only baseline aortic valve calcium and not Lp(a) is associated with disease progression [12]. Overall, while elevated Lp(a) is now recognized as an important factor in the development of AVS, its precise role in disease progression remains less clearly defined. The heterogeneity of findings across studies may, at least in part, reflect the use of different endpoints—such as hemodynamic parameters versus changes in aortic valve calcium score—which may capture distinct aspects of the disease process. Clarifying this relationship, particularly in patients with mild-to-moderate disease, will be essential to understanding whether Lp(a)-lowering interventions could modify the natural history of aortic stenosis.

Taken together, these findings suggest that lipoprotein(a) primarily acts during the early phases of pathogenesis by promoting valvular calcium deposition, whereas the clinical progression of stenosis is driven by the pre-existing calcium burden. Consequently, quantification of valvular calcium remains the most robust prognostic marker for disease progression, while Lp(a) may be particularly relevant as a risk factor in pre-calcific stages or in patients with already diagnosed aortic stenosis.

Several factors may confound the relationship between Lp(a) levels and aortic valve stenosis, including age, sex, baseline valve calcification, comorbid atherosclerosis, body mass index, and systemic inflammation. The association appears weaker in older or female populations and is largely influenced by baseline calcium burden, which remains the strongest predictor of disease progression [35,36]. Shared cardiovascular risk factors further obscure causality, while inflammation shows little modifying effect. Overall, Lp(a) seems more closely linked to the onset of calcification than to its progression, underscoring the need for careful adjustment of confounders in both observational and interventional studies.

Building upon this pathophysiological rationale, Lp(a) has emerged as a potential contributor to the degeneration of biological valve prostheses, promoting bioprosthetic valve mineralization, through the same mechanisms as in the native valve. However, data on short-term progression remain conflicting, and some studies have not demonstrated a significant association over limited follow-up, suggesting a possible time- or subgroup-dependent effect [37].

## 4. Measurement of Lp(a) and Clinical Guidelines

Based upon the previous paragraphs, the availability of a reliable Lp(a) assessment represents a crucial tool to improve clinical practice, but the complexity of this molecule makes its measurement problematic. The composition of Lp(a) varies depending on genotype, cholesterol, cholesterol esters, phospholipids, apo B100, and apoA [38]. In particular, a major challenge for the precise measurement of Lp(a) is represented by the large heterogeneity in apo(a) size between subjects but also within the same individual for the inheritance of two different apo(a) alleles.

The polyclonal antibodies commonly used against apo(a) cross-react with the multiple KIV2 repeats, which lead to overestimation of Lp(a) plasma concentration in individuals with large isoforms and underestimation of those with small isoforms, with consequence in risk stratification. Recent research has highlighted a consistent positive relationship between high levels of Lp(a) measured though ELISA assay methods and cardiovascular disease [13]. In fact, this method uses monoclonal antibodies targeting an epitope in KIV9, providing a valid approach comparable to liquid chromatography/mass spectrometry and it shows. Lately, a new assay based on magnetic particle–based isolation of Lp(a) could be included in clinical practice in the future [35].

Current recommendations encourage to report the results of Lp(a) protein measurement in nanomoles per liter, reflecting a precise and selective interaction of antibody with apo(a). Historically, Lp(a) has been expressed in mass units (mg/dL) encompassing the mass of the whole particle [39]. That is methodologically improper because what is measured by immunoassays is the protein component of Lp(a) and not its lipid and carbohydrate content. Consequently, conversion from one to another is strongly discouraged, as all conversion factors are inherently isoform-dependent [36]. However, both mass and molar assays have been associated with prognostic value in predicting the risk for major adverse cardiovascular events.

In the last few years, guidelines of international societies have incorporated Lp(a) testing into their recommendations and current practice, highlighting the strong link between its dosage and atherosclerotic cardiovascular disease (ASCVD) risk. The ESC and the CCS (Canadian Cardiovascular Society) recommend all adults be tested for elevated Lp(a) at least once in their lifetime; thus, the ACC/AHA (American College of Cardiology/American Heart Association) recommends to screen intermediate- or high-risk individuals, subjects with familial hypercholesterolemia, those with a family history of cardiovascular disease, or those poorly responsive to other LDL-C-lowering therapies [35,40,41]. The HEART-UK in a dedicated consensus suggests Lp(a) plasma level evaluation in adults with a personal or family history of premature ASCVD, first-degree relatives who have Lp(a) levels >200 nmol/L, patients with familial hypercholesterolemia, or in those with calcific AVS [42].

There is no generalized consensus on Lp(a) risk thresholds: ≥50 mg/dL (or ≥125 nmol/L) is an accepted target in ACC/AHA guidelines; thus, ≥50 mg/dL (or ≥100 nmol/L), considering primary prevention, is a valuable target in the CCS ones [40].

The ESC/EAS 2022 panel consensus suggests a pragmatic approach with Lp(a) cut-offs to ‘rule out’ (<30 mg/dL or <75 nmol/L) or ‘rule-in’ (>50 mg/dL or >125 nmol/L) risk. The interim gray zone (30–50 mg/dL; 75–125 nmol/l) is relevant when considering Lp(a)-attributable risk in the presence of other risk factors and in risk stratification [42]. The cut-off threshold changes on >180 mg/dL (>430 nmol/L) when considering equivalent to heterozygous familial hypercholesterolemia [35,43]. However, interpretation of these thresholds should be approached with caution, as variability among available assays and lack of complete standardization can influence measured concentrations and, consequently, individual risk classification

## 5. Non-Specific Pharmacological Approaches Impacting Lp(a)

Only during last decade pharmacological approaches have been developed specifically to target Lp(a), and many of these are currently under investigation. However, established lipid-lowering therapies have shown to influence Lp(a) plasma level concentration. This section will describe the impact on Lp(a) of lipid-lowering drug commonly used in clinical practice (Table 1).

### 5.1. Statins

Alongside the growing adoption of healthy lifestyles and the increasing recognition of their importance in overall well-being, statins have played a key role in reducing the prevalence of elevated LDL-C levels [19,44]. While their LDL-C-lowering effects are well established, there remains considerable uncertainty regarding the extent that statins influence Lp(a) levels. Although the literature is controversial, it was noted as early as 1989 that lovastatin causes a dose-dependent increase in Lp(a) [45]. The mechanisms underlying the selective increase in Lp(a) levels during statin therapy in carriers of a small apo(a) are unclear and require further mechanistic studies. Cell culture studies revealed a time- and dose-dependent, statin-mediated increase in LPA mRNA expression and apo(a) production, suggesting the mechanism is in part related to increased Lp(a) production [46].

A subject-level meta-analysis, which has included 5256 patients (1371 on placebo and 3885 on statin) from six randomized trials (three statin vs. placebo trials, and three statin vs. statin trials), has revealed that statins significantly increase plasma Lp(a) levels [47]. In the statin-vs.-placebo pooled analysis, the ratio of geometric means [95% confidence interval (CI)] for statin to placebo is 1.11 (1.07–1.14) (*p*  <  0.0001), with ratio >1 indicating a higher increase in Lp(a) from baseline in statin vs. placebo. In the statin vs. statin pooled analysis, the ratio of geometric means (95% CI) for atorvastatin to pravastatin is 1.09 (1.05–1.14) (*p*  <  0.0001). The mean percent change from baseline ranged from 11.6% to 20.4% in the pravastatin group and 18.7% to 24.2% in the atorvastatin group; this finding further underscores that the effect is correlated with statin potency, exhibiting greater magnitude with higher-intensity agents.

Interesting, the adverse consequences of increased Lp(a) levels post-statin regimen may play a role in the residual risk in patients on statin therapy, so they should be evaluated in future studies.

Beyond statin therapy, additional lipid-lowering agents have the potential to modulate Lp(a) synthesis and circulating concentrations, thereby contributing to the attenuation of adverse cardiovascular outcomes associated with their use.

### 5.2. Ezetimibe

Ezetimibe has demonstrated to exert anti-inflammatory effects, and there is evidence suggesting that Lp(a) plays as an acute-phase reactant, with its biosynthesis upregulated in the setting of inflammation [46,48]. Therefore, ezetimibe may also influence Lp(a) production in this way. The involvement of the LDLR in the catabolism and clearance of plasma Lp(a) has been proposed, and ezetimibe has been reported to enhance the statin-induced upregulation of LDLR gene expression [49]. Taken together, these findings support the plausibility that the clinical benefits of ezetimibe, as demonstrated in the IMPROVE-IT trial (Improved Reduction of Outcomes: Vytorin Efficacy International Trial), may be attributable, at least in part, to reductions in Lp(a) levels [50]. A meta-analysis including seven randomized controlled trials (RCTs) with a total of 2337 patients reported that treatment with ezetimibe 10 mg significantly reduced plasma Lp(a) concentrations in patients with primary hypercholesterolemia by −7.06% (95% CI, −11.95 to −2.18; *p* = 0.005) compared with placebo [51]. According to current evidence, this reduction lacks clinical relevance. Further studies are needed to elucidate the underlying mechanisms of ezetimibe and assess its efficacy in combination with other Lp(a)-lowering agents.

### 5.3. PCSK9i

The class of PCSK9i stands out in the landscape of lipid-lowering drugs for its power in LDL-C reduction, as well as its favorable effects on atherosclerotic plaque [52,53]. Extending the protective effects of PCSK9i on cardiovascular outcomes is the evidence supporting their ability to reduce Lp(a) levels. These findings have emerged from post hoc analyses of clinical trials in which each PCSK9i were not specifically designed to assess their impact on Lp(a), yet the results appear consistent across the drug class, including both monoclonal antibodies and siRNA-based agents.

In fact, PCSK9i are among the few lipid-lowering drugs which offer Lp(a)-lowering effects and may provide further clinical utility: in vivo and in vitro data support the hypothesis that the additional upregulation of LDLR activity by PCSK9i also increases the clearance of Lp(a) [54].

In a prespecified post hoc analysis of the FOURIER trial (Further Cardiovascular Outcomes Research With PCSK9 Inhibition in Subjects With Elevated Risk) Lp(a) was measured at baseline in 25,096 patients enrolled [55]. At 48 weeks, evolocumab significantly reduced Lp(a) by a median of 26.9% (6.2–46.7%). The percent change in Lp(a) and LDL-C at 48 weeks in patients taking evolocumab was moderately positively correlated (r = 0.37; 95% CI, 0.36–0.39; *p* < 0.001). Evolocumab also reduced the risk of coronary heart disease death, myocardial infarction, or urgent revascularization by 23% (hazard ratio, 0.77; 95% CI, 0.67–0.88) in patients with a baseline Lp(a) > median, and by 7% (hazard ratio, 0.93; 95% CI, 0.80–1.08; P interaction = 0.07) in those ≤ median.

The Program to Reduce LDL-C and Cardiovascular Outcomes Following Inhibition of PCSK9 In Different Populations (PROFICIO) was a pooled analysis of 3278 patients on different background lipid-lowering therapies, which showed how bi-weekly and monthly doses of evolocumab statistically significant reduced Lp(a) at Week 12 vs. control (*p* < 0.001) with a percent reduction of 24.7% and 21.7%, respectively [56]. The greater percent reduction in Lp(a) observed in patients who had lower levels of LDL-C compared with those with higher levels is supportive of the hypothesis that Lp(a) competes with LDL-C for the LDLR/apoB receptor.

Similar results have been reported using the other PCSK9i monoclonal antibody, Alirocumab.

An analysis of pooled data from the phase 3 ODYSSEY program, including 4915 patients with hypercholesterolemia from 10 phase 3 studies, showed the use of Alirocumab was associated with a reduction in Lp(a)between 23% and 29% according to the drug regimen (alirocumab 75 mg or 150 mg every 2 weeks) [57].

In conclusion, the available small interfering RiboNucleic Acid (siRNA) targeting PCSK9, Inclisiran, have confirmed Lp(a)-reducing effect, as well as the other PCSK9i. In particular, at day 180, the ORION-1 study reported an Lp(a) reduction ranging from 14% to 18% in the single-dose groups and from 15% to 26% with 2-dose regimens [58]. Moreover, Inclisiran therapy was shown to be associated with Lp(a) reduction by 13.5% among patients with heterozygous familial hypercholesterolemia in the ORION-9 trial, by 21.9% in patients with established ASCVD in the ORION-10 trial, and by 18.6% among patients with ASCVD or an ASCVD equivalent in ORION-11 [59,60].

### 5.4. Bempedoic Acid

Bempedoic acid, recently approved as the third oral lipid-lowering agent after statins and ezetimibe, reduces apoB and LDL-C by ~20%, depending on concomitant therapy. Current evidence indicates minimal impact on Lp(a). Acting via ATPCLY (Adenosine triphosphate citrate lyase) inhibition, upstream of Hydroxymethylglutaryl-coenzyme A reductase, its effect on Lp(a) appears comparable to that of statins [61,62].

### 5.5. Omega-3 Fatty Acids

Omega-3 fatty acids (ω-3FAs), particularly eicosapentaenoic acid and docosahexaenoic acid, exert lipid-modulating and anti-inflammatory effects through mechanisms involving reduced hepatic Very Low-Density Lipoprotein synthesis, enhanced clearance of triglyceride-rich particles, and modulation of inflammatory signaling pathways. Their impact on Lp(a), however, remains poorly defined. In a pilot study of 12 patients with stable CAD and elevated Lp(a) (>0.5 g/L) receiving background lipid-lowering therapy, high-dose ω-3FAs (3.6 g/day) significantly reduced Lp(a) plasma levels by 5% (*p* < 0.01), concomitant with a 17% reduction in triglycerides. These findings suggest a modest but measurable effect of ω-3FAs on Lp(a)-associated cardiovascular risk [63].

## 6. Specific Lp(a)-Lowering Therapies: Mechanisms and Trials

Over the course of the last decade, the evidence establishing Lp(a) as a newer risk factor, pushed the clinical development of novel, specific therapeutic strategies to reduce Lp(a) plasma concentration (Table 1). Different drugs are under investigation, and many ongoing trials will provide evidence about the effect of specific Lp(a) drugs on cardiovascular clinical outcomes (Table 2).

### 6.1. Antisense Oligonucleotides

Pelacarsen (TQJ230) is a next-generation antisense oligonucleotide designed to selectively inhibit the synthesis of apo(a), a key structural component of Lp(a) [64,65,66]. The molecule incorporates 2′-O-methoxyethyl (2′-MOE) modifications and a triantennary N-acetylgalactosamine (GalNAc) conjugate, which enhances binding to the asialoglycoprotein receptor on hepatocytes and promotes targeted uptake [64,65,66]. Once inside the nucleus, hybridization to apo(a) mRNA triggers RNase H–mediated degradation, thereby blocking protein translation) [64,65,66]. This mechanism results in a marked reduction in Lp(a) levels, independent of LPA genotype and apo(a) isoform size, and is accompanied by parallel decreases in apolipoprotein B, oxidized phospholipids, and corrected LDL-C, supporting its action on atherothrombotic and pro-calcific pathways [67].

Across early phase 1 and phase 1/2a studies in adults with elevated Lp(a), pelacarsen produced dose-dependent reductions of up to ~90 to 92 percent, with effects sustained for up to four months after the last dose and no serious adverse events [65,68]. In a randomized phase 2 study in 286 patients with established ASCVD and Lp(a) at least 150 nmol/L, regimens from 20 mg weekly to 60 mg every four weeks produced reductions of 72 to 80 percent versus about 6 percent with placebo, with a good tolerability characterized by mild injection-site reactions, and persistence of effect for months; parallel decreases in oxidized phospholipids, apolipoprotein B, and corrected LDL-C accompanied Lp(a) lowering [64].

Currently, a phase 3 outcomes trial, Lp(a)HORIZON (NCT04023552), is ongoing. The study is randomizing 8323 patients with prior myocardial infarction, ischemic stroke, or symptomatic peripheral artery disease and Lp(a) ≥ 70 mg/dL to pelacarsen 80 mg every four weeks or placebo on top of optimized lipid-lowering therapy [69]. The primary endpoint is a composite of cardiovascular death, nonfatal myocardial infarction, nonfatal ischemic stroke, or urgent coronary revascularization requiring hospitalization, and primary completion is anticipated in 2026.

In parallel, an ongoing dedicated phase 2 trial, Lp(a) FRONTIERS CAVS (NCT05646381), is evaluating whether monthly pelacarsen 80 mg slows the progression of calcific AVS in approximately 502 adults aged 50 to 80 years with mild or moderate disease and Lp(a) at least 175 nmol/L. This randomized, double-blind, placebo-controlled study uses change in peak aortic jet velocity and computed tomography aortic valve calcium as co-primary endpoints, with estimated completion in March 2030.

### 6.2. siRNAs

siRNAs are chemically synthesized and equipped with a triantennary N-acetylgalactosamine ligand that directs receptor-mediated uptake by hepatocytes through the asialoglycoprotein receptor. Within the cytoplasm, they recruit the RNA-induced silencing complex to cleave apo(a) mRNA, thereby suppressing hepatic production of Lp(a) [70,71]. Three dedicated agents are in clinical development against Lp(a): olpasiran (AMG890), zerlasiran (SLN360), and lepodisiran (LY3819469). Across phase 1 and phase 2 studies, subcutaneous regimens produced dose-dependent reductions from about 40 percent at lower doses to approximately 99 percent at higher doses, with durable effects and a generally favorable safety profile [72,73,74,75,76,77,78].

In this context, olpasiran showed clear dose-dependent Lp(a) reductions in phase 1 studies and in the phase 2 OCEAN(a) DOSE (Olpasiran Trials of Cardiovascular Events and Lipoprotein(a) Reduction–Dose Finding Study) trial of 281 patients with ASCVD and baseline Lp(a) above 150 nmol/L, where dosing every 12 or 24 weeks achieved large and consistent percentage reductions, with overall adverse event rates similar to placebo although hypersensitivity and injection-site reactions were more frequent on active treatment [72,74,75]. However, OCEAN(a) DOSE did not assess clinical endpoints and had limited safety follow-up. The ongoing randomized, double-blind, placebo-controlled phase 3 OCEAN(a) Outcomes (Olpasiran Trials of Cardiovascular Events and Lipoprotein(a) Reduction trial (NCT05581303) is testing whether olpasiran reduces coronary heart disease death, myocardial infarction, or urgent coronary revascularization in 6000 patients with Lp(a) at least 200 nmol/L, with estimated primary completion in December 2026. Simultaneously, lepodisiran produced a dose-dependent and sustained Lp(a) lowering in a randomized phase 1 dose escalation study in adults without ASCVD and baseline Lp(a) at least 75 nmol/L, achieving a maximal reduction near 97 percent with a single high dose [79]. In the phase 2 ALPACA trial, 320 adults with Lp(a) at least 175 nmol/L were randomized to several subcutaneous dose regimens of lepodisiran or placebo. The 400 mg regimen, analyzed as pooled groups, delivered a placebo-adjusted, time-averaged reduction in Lp(a) of 93.9 percent from day 60 to day 180, with durable lowering through day 54013. No serious adverse events were judged related to treatment, and injection-site reactions were dose-dependent and generally mild, occurring in up to 12 percent at the highest dose. Building on these findings, the randomized, double-blind, placebo-controlled phase 3 ACCLAIM Lp(a) trial (A Study to Investigate the Effect of Lepodisiran on the Reduction of Major Adverse Cardiovascular Events in Adults With Elevated Lipoprotein(a)) (NCT06292013) is testing whether lepodisiran reduces major adverse cardiovascular events in approximately 16,700 adults with Lp(a) at least 175 nmol/L who either have established ASCVD or are at high risk for a first cardiovascular event; primary completion is estimated for March 2029.

Finally, zerlasiran showed potent Lp(a) lowering in the randomized phase 1 APOLLO study of adults, with baseline Lp(a) at least 150 nmol/L, and no history of ASCVD, with single subcutaneous doses up to 600 mg achieving reductions approaching 98 percent and no treatment-related serious adverse events [80]. In the phase 2 ALPACAR-360 trial, 178 patients with stable ASCVD and baseline Lp(a) at least 125 nmol/L were assigned to three dosing strategies (450 mg or 300 mg every 24 weeks for 2 doses or 300 mg every 16 weeks for 3 doses) or placebo. Time-averaged reductions to week 36 exceeded 80 percent in all active arms, with median on-treatment reductions around 90 to 96 percent at week 36. Injection-site reactions were generally mild, and no serious adverse events were judged related to treatment [81]. A phase 3 cardiovascular outcomes trial has not yet started.

Despite epidemiologic and genetic evidence linking elevated Lp(a) to the presence, development, and progression of calcific AVS, most recently published and ongoing trials of specific Lp(a)-lowering therapies are focused on atherosclerotic cardiovascular outcomes rather than valve-specific endpoints, leaving it uncertain whether substantial Lp(a) reduction slows established calcific AVS or delays the need for intervention [82]. In this context, the ongoing phase 2 Lp(a) FRONTIERS CAVS study is, to date, the only trial specifically focused on valve disease progression. Pending these results, application to calcific AVS should be regarded as investigational; should a benefit be demonstrated, the therapeutic window is most likely early in the disease course, before advanced calcification predominates [83].

Finally, ASO and siRNA differ in magnitude and duration of Lp(a) reduction. siRNA-based agents generally achieve deeper and more sustained lowering (up to ~98%) with infrequent dosing intervals (every 3–12 months), whereas pelacarsen reduces Lp(a) by up to ~80% and requires monthly administration. Both classes employ N-acetyl-galactosamine conjugation for selective hepatocyte delivery and exhibit favorable safety profiles, with mild injection-site reactions being the most frequent adverse events. Overall, siRNAs offer longer dosing intervals and convenience, while pelacarsen currently has the most advanced clinical outcome data among RNA-targeted therapies.

## 7. Conclusions

AVS is the most common valve disease in the elderly, with poor prognosis and only interventional approaches currently available. To date, no drug has been shown to halt the natural progression of AVS. Years of dedicated research have deepened our understanding of mechanisms promoting development and progression of this valvular disease, and the discovery of a pathogenesis similar to the one of atherosclerosis have underlined the importance of dyslipidemia, inflammation, and calcification. The discovery of a Lp(a) link to cardiovascular disease, with particular attention to AVS, has been a keypoint in the field of cardiovascular pharmacotherapy. Despite the extensive literature raising Lp(a) as an additional cardiovascular risk factor, and the efforts to identify the best method to assess its plasma concentration, Lp(a) measurement has been not yet widely implemented in clinical practice, since, apparently, it is not an actionable therapeutic target. Nevertheless, currently used lipid-lowering drugs have been shown to impact Lp(a) level in different ways. Among these, the PCSK9i, both monoclonal antibodies and siRNA, exert the most desirable effects on Lp(a) offering a further effect on cardiovascular prevention, even if these agents have not been specifically tested for this purpose. Emerging molecules have been developed to target Lp(a) through different mechanisms of action, and clinical trials are now ongoing. Thus, the impact of Lp(a) reduction on clinical outcomes will finally be clearer but more clinical studies should be awaited to understand the clinical implications in the AVS setting.

In the near future, Lp(a)-lowering therapies could be integrated into AVS management as disease-modifying agents, particularly for patients with mild or moderate calcific stenosis and elevated Lp(a) levels. These individuals may represent the subgroup most likely to benefit from early pharmacological intervention, before advanced valve calcification occurs. To conclude, routine practice is expected to change soon, once therapies specifically targeting Lp(a) become available, providing a pharmacological opportunity for AVS treatment.

## Figures and Tables

**Table 1:** Effect of specific and non-specific lipid-lowering drugs on plasma Lp(a) concentration.

Drug	Mechanism of Action	Effect on Lp(a)
**Non-Specific Lp(a)-lowering therapies**		
**Statins**	HMG-CoA reductase inhibition; increased LPA mRNA expression and apo(a) production	Increase ~12–24% (dose and potency dependent)
**Ezetimibe**	Inhibition of intestinal cholesterol absorption; possible LDLR upregulation and anti-inflammatory effects	Decrease ~7%
**PCSK9-i**	Increased LDLR activity and Lp(a) clearance	Decrease 14–30%
**Bempedoic acid**	ATP-citrate lyase inhibition	Minimal effect
**Omega-3 fatty acids** **(EPA/DHA)**	Reduced hepatic VLDL synthesis, increased clearance of TG-rich particles, anti-inflammatory effects	Decrease ~5% (preliminary data, small sample)
**Specific Lp(a)-lowering therapies**		
**Pelacarsen**	Antisense oligonucleotide targeting hepatic LPA mRNA, inhibiting apo(a) synthesis	Decrease up to ~80% (dose-dependent, monthly SC dosing)
**Olpasiran**	GalNAc-conjugated siRNA inhibiting LPA mRNA translation in hepatocytes	Decrease up to ~95–98% (quarterly or biannual dosing)
**Zerlasiran**	GalNAc-conjugated siRNA targeting LPA mRNA	Decrease ~90–96% (every 6–12 months)
**Lepodisiran**	Long-acting GalNAc-siRNA targeting LPA mRNA	Decrease up to 98% sustained ≥48 weeks after a single dose

**Abbreviations:** ASO, Antisense Oligonucleotide; siRNA ATP, Adenosine TriPhosphate; DHA, Docosahexaenoic Acid; EPA, Eicosapentaenoic Acid; GalNAc, N-acetylgalactosamine; HMG-CoA, 3-idrossi-3-metilglutaril-coenzima A; LDL-R, Low-Density Lipoprotein receptor; Lp(a), Lipoprotein(a); PCSK9-I, Proprotein Convertase Subtilisin/Kexin type 9 inhibitor; SC, Subcutaneous; siRNA, Small Interfering RNA.

**Table 2:** Ongoing phase 3 trials of specific drugs targeting lipoprotein (a).

Trial	Lp(a)HORIZON	OCEAN(a)–Outcomes Trial	ACCLAIM Lp(a)
**Sample**	N = 8323	N = 7297	N = 12,500
**Population**	Patients with established ASCVD and Lp(a) > 175 nmol/L (70 mg/dL)	Patients with Lp(a) > 200 nmol/L, a history of ASCVD (defined as either a previous type 1 MI or previous revascularization with PCI) and at least 1 prespecified risk-enhancing feature	Patients with Lp(a) > 175 nmol/L and at high risk of cardiovascular events or with established ASCVD
**Investigational** **drug**	Pelacarsen 80 mg, injected subcutaneously once per month	Olpasiran, injected subcutaneously every 12 weeks	Lepodisiran, injected subcutaneously
**Primary** **outcome(s)**	Time to first MACE (cardiovascular death, nonfatal MI, nonfatal stroke, or urgent coronary revascularization requiring hospitalization)	Time to first MACE (death from coronary artery disease, MI, or urgent coronary revascularization)	Time to first MACE (cardiovascular death, MI, stroke, or urgent coronary revascularization)
**Expected completion date (month/year)**	May 2025	December 2026	March 2029

**Abbreviations:** ASCVD indicates atherosclerotic cardiovascular disease; Lp(a), lipoprotein(a); MACE, major adverse cardiovascular events; MI, myocardial infarction; PCI, percutaneous coronary intervention.

## Data Availability

The original contributions presented in this study are included in the article. Further inquiries can be directed to the corresponding author(s).
